# Long-term survivals of immune checkpoint inhibitors as neoadjuvant and adjuvant therapy in dMMR/MSI-H colorectal and gastric cancers

**DOI:** 10.1007/s00262-024-03764-9

**Published:** 2024-07-05

**Authors:** Zhenghang Wang, Siyuan Cheng, Yanhong Yao, Shengde Liu, Zimin Liu, Ning Liu, Yongdong Jin, Yinjie Zhang, Fei Yin, Guangjie Han, Jingdong Zhang, Qiwei Wang, Dong Yan, Li Wang, Hongxia Lu, Ting Deng, Zhi Ji, Hui Gao, Weijia Fang, Hangyu Zhang, Zhiyu Chen, Jianling Zou, Yong Tang, Chunlei Xu, Jiayi Li, Huajun Qu, Liying Bao, Baoshan Cao, Xicheng Wang, Ting Xu, Yu Sun, Lin Shen, Zhi Peng, Jian Li

**Affiliations:** 1 State Key Laboratory of Holistic Integrative Management of Gastrointestinal Cancers, Beijing Key Laboratory of Carcinogenesis and Translational Research, Department of Gastrointestinal Oncology, Peking University Cancer Hospital and Institute, Beijing, China; 2 Department of Gastrointestinal Oncology, Key Laboratory of Carcinogenesis and Translational Research (Ministry of Education), Peking University Cancer Hospital and Institute, Beijing, China; 3 Department of Oncology, Peking University Cancer Hospital (Lnner Mongolia Campus)/Affiliated Cancer Hospital of Inner Mongolia Medical University, Inner Mongolia Cancer Center, Hohhot, China; 4 Department of Medical Oncology and Radiation Sickness, Peking University Third Hospital, Beijing, China; 5 Department of Oncology, The Affiliated Hospital of Qingdao University, Qingdao, Shandong China; 6 Department of Medical Oncology, Sichuan Cancer Hospital & Institute, Sichuan Cancer Center, School of Medicine, University of Electronic Science and Technology of China, Chengdu, Sichuan China; 7 Department of Gastroenterology and Hepatology, The Fourth Hospital of Hebei Medical University, Shijiazhuang, Hebei China; 8 Medical Oncology Department of Gastrointestinal Cancer, Liaoning Cancer Hospital & Institute, Cancer Hospital of China Medical University, Shenyang, China; 9 Department of Oncology, Beijing Luhe Hospital Affiliated to Capital Medical University, Beijing, China; 10 Department of Gastroenterology, Shanxi Province Cancer Hospital/ Shanxi Hospital Affiliated to Cancer Hospital, Chinese Academy of Medical Sciences/Cancer Hospital Affiliated to Shanxi Medical University, Taiyuan, Shanxi China; 11 Department of Gastrointestinal Oncology, Tianjin Medical University Cancer Institute and Hospital, National Clinical Research Center for Cancer, Key Laboratory of Cancer Prevention and Therapy, Tianjin’s Clinical Research Center for Cancer, Tianjin, China; 12 Department of Oncology Rehabilitation, Zhengzhou Central Hospital Affiliated to Zhengzhou University, Zhengzhou, Henan China; 13 Department of Medical Oncology, First Affiliated Hospital, School of Medicine, Zhejiang University, Hangzhou, Zhejiang China; 14 Department of Gastrointestinal Medical Oncology, Fudan University Shanghai Cancer Center, Shanghai, China; 15 Department of Digestive Internal Medicine, The Affiliated Tumor Hospital of Xinjiang Medical University, Urumqi, Xinjiang China; 16 Department of Medical Oncology, The First Affliated Hospital of Xiamen University, School of Medicine, Xiamen University, Xiamen, China; 17 Department of Medical Oncology, Manzhouli People’s Hospital, Manzhouli, Inner Mongolia China; 18 State Key Laboratory of Holistic Integrative Management of Gastrointestinal Cancers, Beijing Key Laboratory of Carcinogenesis and Translational Research, Department of Pathology, Peking University Cancer Hospital & Institute, Beijing, China; 19 Department of Medical Oncology, The Affiliated Yantai Yuhuangding Hospital of Qingdao University, Yantai, Shandong China

**Keywords:** Microsatellite instability-high, Deficient mismatch repair, Gastrointestinal neoplasm, Perioperative treatment, Immunotherapy

## Abstract

**Background:**

The long-term survival benefit of immune checkpoint inhibitors (ICIs) in neoadjuvant and adjuvant settings is unclear for colorectal cancers (CRC) and gastric cancers (GC) with deficiency of mismatch repair (dMMR) or microsatellite instability-high (MSI-H).

**Methods:**

This retrospective study enrolled patients with dMMR/MSI-H CRC and GC who received at least one dose of neoadjuvant ICIs (neoadjuvant cohort, NAC) or adjuvant ICIs (adjuvant cohort, AC) at 17 centers in China. Patients with stage IV disease were also eligible if all tumor lesions were radically resectable.

**Results:**

In NAC (*n* = 124), objective response rates were 75.7% and 55.4%, respectively, in CRC and GC, and pathological complete response rates were 73.4% and 47.7%, respectively. The 3-year disease-free survival (DFS) and overall survival (OS) rates were 96% (95%CI 90–100%) and 100% for CRC (median follow-up [mFU] 29.4 months), respectively, and were 84% (72–96%) and 93% (85–100%) for GC (mFU 33.0 months), respectively. In AC (*n* = 48), the 3-year DFS and OS rates were 94% (84–100%) and 100% for CRC (mFU 35.5 months), respectively, and were 92% (82–100%) and 96% (88–100%) for GC (mFU 40.4 months), respectively. Among the seven patients with distant relapse, four received dual blockade of PD1 and CTLA4 combined with or without chemo- and targeted drugs, with three partial response and one progressive disease.

**Conclusion:**

With a relatively long follow-up, this study demonstrated that neoadjuvant and adjuvant ICIs might be both associated with promising DFS and OS in dMMR/MSI-H CRC and GC, which should be confirmed in further randomized clinical trials.

**Supplementary Information:**

The online version contains supplementary material available at 10.1007/s00262-024-03764-9.

## Background

Microsatellite instability-high (MSI-H) subtypes make up approximately 15–20% of all gastrointestinal (GI) cancers. Due to the deficiency of mismatch repair (dMMR), these tumors are characterized by high levels of tumor mutation burden and abundant tumor-infiltrating lymphocytes [1–3]. Researches have shown that immune checkpoint inhibitors (ICIs) could be used as standard therapy of unresectable or metastatic dMMR/MSI-H gastric cancer (GC) and colorectal cancer (CRC) [4–10].

Taken into consideration the promising efficacy of ICIs in the palliative setting, the role of ICIs has also been investigated in perioperative settings. Overall, the use of neoadjuvant ICIs is safe and has little impact on sequent surgery [11–15]. However, these data all come from PD1 antibody with or without cytotoxic T lymphocyte-associated antigen-4 (CTLA4) antibody in prospective clinical trials, and could not reflect the clinical practice in the real world, where chemotherapy might be combined with ICIs. And also, the safety profile with adjuvant ICIs has not been explored.

Regarding to the efficacy, some small sample studies have shown that neoadjuvant ICIs could lead to relatively high pathologically complete response (pCR) rates [11–15]. However, it remains unclear whether the high pCR rates can translate into subsequent survival benefits. As for adjuvant setting, patients with dMMR/MSI-H gastric cancer (GC) seemed to not benefit from adjuvant chemotherapy [16]. And around 20–25% of patients with stage III dMMR/MSI-H colorectal cancer (CRC) suffered from recurrence even after adjuvant chemotherapy [17]. It is widely discussed whether adjuvant ICIs should be applied in patients not treated with preoperative ICIs. In China, a small proportion of patients decided to receive adjuvant ICIs with or without chemotherapy after being fully informed of the potential pros and cons in a patient-doctor shared decision-making process. However, no study reported the survival data of adjuvant ICIs in dMMR/MSI-H GI cancers so far.

In order to investigate the long-term survivals of neoadjuvant and adjuvant ICIs in dMMR/MSI-H GC and CRC, we retrospectively collected and analyzed the efficacy data from 85 patients enrolled in a prospective observational study (NCT04640103) and other 87 patients retrieved from medical systems from 17 centers.

## Methods

### Patients Selection and study design

Patients from a prospective observational real-world study (NCT04640103) and patients derived from medical systems from 17 centers were combined together for retrospective efficacy analysis. Main inclusion criteria were: (1) age ≥ 18 years old, (2) confirmed by pathology as gastric cancer (including esophagogastric junction adenocarcinoma) or colorectal adenocarcinoma, (3) dMMR by immunohistochemistry or MSI-H confirmed by polymerase chain reaction (PCR) or next generation sequencing (NGS), and (4) received at least one dose of ICIs with or without chemotherapy or targeted drugs in neoadjuvant or adjuvant setting. (5) patients with stage IV disease were also included if all tumor lesions were assessed as resectable (6) patients treated with neoadjuvant ICIs with or without adjuvant therapy were included in the neoadjuvant cohort (NAC), and those treated with adjuvant ICIs and no any neoadjuvant therapy were included in the adjuvant cohort (AC). Patients were excluded if detailed medical records or follow-up were not available. Patients with proficient mismatch repair (pMMR) or unknown MMR status but MSI-H were included, while those with dMMR and microsatellite stability (MSS) were excluded.

This study was approved by the ethics committee of Beijing Cancer Hospital. All patients had signed informed consent for data collection.

### Assessments and outcomes

The NAC patients were evaluated by imaging examination every 6–8 weeks after the initial of treatment (including chest, abdominal and pelvic CT, as well as pelvic and liver MRI if necessary). The time for operation and adjuvant therapy were decided by doctors in charge. Patients were followed up according to local guidelines. The criterion for terminating follow-up is death for any reason.

The duration of neoadjuvant therapy was defied as the time from the first dose of neoadjuvant treatment to surgery or clinical complete response (cCR) (for patients who achieved cCR did not receive surgery) or the last dose (for patients who did not achieve cCR and did not receive surgery), and the duration of adjuvant therapy was defied as the time from the first dose of adjuvant treatment to the last dose. Efficacy outcomes included best response (for NAC), pCR rate (for patients receiving surgery in NAC), disease-free survival (DFS), overall survival (OS). DFS was defied as the time from surgery or cCR until death or disease recurrence. OS was defied as the time from the first dose of neoadjuvant therapy (for NAC) or surgery (for AC) until death or the date the patient was last known to be alive.

### Statistical analysis

All data were processed using SPSS 26.0 statistical software. Kaplan Meier curves was created by GraphPad Prism 9.5.1. Continuous variables were represented by a median (range). The classified variables were tested by chi-square test or Fisher exact test. A binary logistic regression model was performed to identify independent prognostic factors of pCR in NAC. The difference was statistically significant with *P* < 0.05. Survival rates were estimated by life table analysis.

## Results

**Fig. 1:**
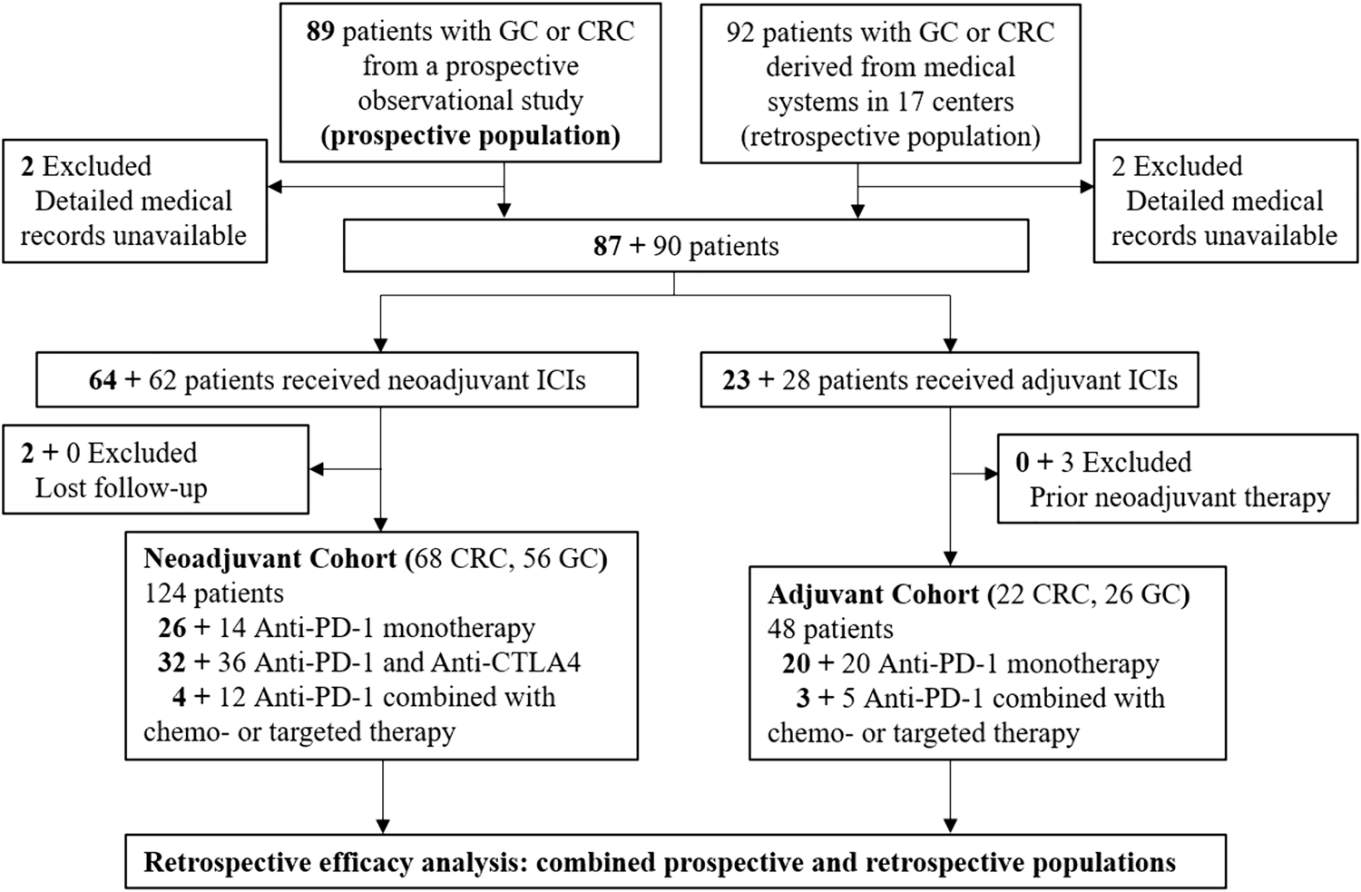
Trial profile, Bold and Gray refers to prospective population and retrospective population, respectively. CRC: colorectal cancer; GC: gastric cancer; ICI: immune checkpoint inhibitor

A total of 181 patients were screened in this study, with 89 from our prospective observational trial from January 2020 to February 2024 and 92 from 17 centers from September 2017 to July 2023 (Fig. 1), and finally 172 patients were included in the study, with 124 in NAC and 48 in AC.

### Neoadjuvant cohort

#### Patient characteristics

On April 10, 2024, at data cut-off, the median follow-up time was 29.4 months (4.3 to 79.4 months) for the CRC and 33.0 months (3.2 to 68.9 months) for GC.

The median age for CRC was 45.5 years (ranging from 19 to 71 years), younger than that for GC (median 65.5 years, ranging from 36 to 81 years). More CRC tumors (77.2%) were defined as MSI-H by PCR or NGS than GC. There was no significant difference in other clinical features between CRC and GC (Table 1). As for treatment regimen, most patients, especially those with GC, received anti-PD1 antibody combined with chemo- or targeted therapy as neoadjuvant treatment (Supplementary Tables 1 and 2). The median duration of neoadjuvant treatment was 4 months (ranging from 1 to 25 months) and 3 months (ranging from 1 to 25 months) for CRC and GC, respectively. Observation was the most preferred strategy after surgery, followed by anti-PD1 antibody monotherapy (Supplementary Tables 1 and 2). The median duration of adjuvant treatment was 4 months (ranging from 1 to 26 months) and 4 months (ranging from 1 to 12 months) for CRC and GC, respectively.

**Table 1:** Clinical characteristics of Neoadjuvant Cohort and Adjuvant Cohort

	Neoadjuvant cohort				Adjuvant cohort			
Clinical factor	Total	Colorectal cancer	Gastric cancer	*P* value	Total	Colorectal cancer	Gastric cancer	*P* value
	(*n* = 124)	(*n* = 68)	(*n* = 56)		(*n* = 48)	(*n* = 22)	(*n* = 26)	
*Gender*								
Female	45(36.3%)	24(35.3%)	21(37.5%)	0.799	18(37.5%)	10(45.5%)	8(30.8%)	0.295
Male	79(63.7%)	44(64.7%)	35(62.5%)		30(62.5%)	12(54.5%)	18(69.2%)	
Median age (years)	57.5	47.5	69.0		56.5	42.5	59.5	
*Age*								
< 60	66(53.2%)	52(76.5%)	14(25.0%)	< 0.001	31(64.6%)	18(81.8%)	13(50.0%)	0.022
≥ 60	58(46.8%)	16(23.5%)	42(75.0%)		17(35.4%)	4(18.2%)	13(50.0%)	
*Tumor location (Colorectal Cancer)*		(*n* = 70^a^)						
Rectum	NA	16(22.9%)	NA		NA	0	NA	
Left-sided colon	NA	13(18.6%)	NA		NA	5(22.7%)	NA	
Right-sided colon	NA	41(58.6%)	NA		NA	17(77.3%)	NA	
*Tumor location (Gastric Cancer)*								
Esophagogastric junction	NA	NA	3(5.4%)		NA	NA	1(3.8%)	
Stomach	NA	NA	53(94.6%)		NA	NA	25(96.2%)	
*MMR status*		(*n* = 70^a^)						
MLH1(-) PMS2(-)	59(46.8%)	22(31.4%)	37(66.1%)	< 0.001	34(70.8%)	15(68.2%)	19(73.1%)	0.243
MSH2(-) MSH6(-)	11(8.7%)	11(15.7%)	0		4(8.3%)	4(18.2%)	0	
PMS2(-)	5(4.0%)	4(5.7%)	1(1.8%)		2(4.2%)	1(4.5%)	1(3.8%)	
MSH6(-)	9(7.1%)	9(12.9%)	0		1(2.1%)	0	1(3.8%)	
Other dMMR pattern	29(23.0%)	16(22.9%)14	13(23.2%)		4(8.3%)	1(4.5%)	3(11.5)	
pMMR^b^	1(0.8%)	1(1.4%)	0		0	0	0	
NA^b^	12(9.5%)	7(10.0%)	5(8.9%)		3(6.3%)	1(4.5%)	2(7.7%)	
*MSI by PCR or NGS*		(*n* = 70^a^)						
MSI-H	80(63.5%)	51(72.9%)	29(51.8%)	0.015	40(83.3%)	16(72.7%)	24(92.3%)	0.119
NA^c^	46(36.5%)	19(27.1%)	27(48.2%)		8(16.7%)	6(27.3%)	2(7.7%)	
*Clinical/Pathological TNM stage*		(*n* = 70^a^)						
II	9(7.1%)	6(8.6%)	3(5.4%)	0.632	9(18.8%)	3(13.6%)	6(23.1%)	0.390
III	87(69.0%)	46(65.7%)	41(73.2%)		30(62.5%)	13(59.1%)	17(65.4%)	
IV	30(23.8%)	18(25.7%)	12(21.4%)		9(18.8%)	6(27.3%)	3(11.5%)	
*Lynch syndrome*								
Yes	6(4.8%)	6(8.8%)	0	0.033	6(12.5%)	5(22.7%)	1(3.8%)	0.001
No	44(35.5%)	20(29.4%)	24(42.9%)		24(50.0%)	5(22.7%)	19(73.1%)	
NA	74(59.7%)	42(61.8%)	32(57.1%)		18(37.5%)	12(54.5%)	6(23.1%)	
*Previous cancer history*								
Yes	15(12.1%)	12(17.6%)	3(5.4%)	0.052	1(2.1%)	0	1(3.8%)	1.000
No	109(87.9%)	56(82.4%)	53(94.6%)		47(97.9%)	22(100.0%)	25(96.2%)	

*NA* not applicable, *MMR* mismatch repair, *dMMR* deficiency of mismatch repair, *MSI* microsatellite instability, *MSI-H* microsatellite instability-high, *pMMR* proficient mismatch repair, *PCR* polymerase chain reaction, *NGS* next generation sequencing

^a^There were two patients diagnosed with both rectal cancer and right-sided colon cancer at the same time, and the tumor location, MMR status, MSI status and TNM staging were recorded separately (Table 2)

^b^These patients were MSI-H

^c^These patients were dMMR

#### Efficacy

All the 124 patients underwent regular imaging evaluations during the neoadjuvant period. Among them, there were two patients with dual primary colorectal cancer (rectal cancer and right-sided colon cancer). They both receive radical resection of all tumors, and therefore, the tumor assessments (radiological and pathological) were recorded separately. The cCR rates were 20.0% and 3.6%, respectively, in CRC and GC. The objective response rates were 75.7% and 55.4%, respectively, in CRC and GC (Table 2).

**Table 2:** Radiographic best response in neoadjuvant cohort

Radiographic best response	Neoadjuvant cohort			*P* value
	Total	Colorectal cancer	Gastric cancer	
	(*n* = 126)	(*n* = 70^a^)	(*n* = 56)	
				0.006
CR	16(12.7%)	14(20.0%)	2(3.6%)	
PR	68(54.0%)	39(55.7%)	29(51.8%)	
SD	40(31.7%)	16(22.9%)	24(42.9%)	
PD	2(1.6%)	1(1.4%)	1(1.8%)	

*CR* complete response, *PR* partial response, *SD* stable disease, *PD* progressive disease, *pCR* pathological complete response

Ninety-nine patients underwent primary tumor resection. The pCR rate was 73.4% and 47.7% for CRC and GC, respectively (Table 3). In multivariate logistic regression analysis and univariate analysis of caner type, gender, age, clinical TNM stage, clinical T stage, neoadjuvant therapy regimen and duration of neoadjuvant therapy, cancer type was the only factor independently significantly associated with pCR rate was found. (Table 3). Notably, the pCR rate in patients with stage IV disease was similar to those without metastasis. And T4 was not a negative predictor of pCR in CRC and GC (Table 3). Twenty-five patients did not receive surgery, including 18 who refused surgery, stopped treatment (10 cCR, 5 partial response [PR], 3 stable disease [SD]) and were in close follow-up and 7 still receiving treatment.

**Table 3:** Chi-square test and multivariate logistic analysis of factors related to pathologic characteristics of surgical patients in neoadjuvant cohort

Pathologic factors	Total				Colorectal cancer				Gastric cancer			
	(*n* = 101)				(*n* = 57^a^)				(*n* = 44)			
	Chi-square test		Multivariate logistic analysis		Chi-square test		Multivariate logistic analysis		Chi-square test		Multivariate logistic analysis	
	pCR rate	*P* value	OR (95%CI)	*P* value	pCR rate	*P* value	OR (95%CI)	*P* value	pCR rate	*P* value	OR (95%CI)	*P* value
*Cancer type*												
CRC	42/57(73.7%)	0.008	Ref									
GC	21/44(47.7%)		0.309(0.105 to 0.911)	0.033								
*Gender*												
Female	24/38(63.2%)	0.900	Ref		13/19(68.4%)	0.523	Ref		11/19(57.9%)	0.239	Ref	
Male	39/63(61.9%)		0.898(0.341 to 2.360)	0.827	29/38(76.3%)		1.509(0.356 to 6.407)	0.577	10/25(40.0%)		0.800(0.185 to 3.449)	0.764
*Age*												
< 60	38/59(64.4%)	0.618	Ref		34/45(75.6%)	0.713	Ref		4/14(28.6%)	0.111	Ref	
≥ 60	25/42(59.5%)		1.764(0.625 to 4.978)	0.284	8/12(66.7%)		0.941(0.197 to 4.490)	0.939	17/30(56.7%)		3.779(0.711 to 20.098)	0.119
*Clinical TNM stage*												
IV	16/27(59.3%)	0.766	Ref		12/17(70.6%)	0.783	Ref		4/10(40.0%)	0.858	Ref	
III	43/69(62.3%)		1.389(0.471 to 4.097)	0.552	27/37(73.0%)		1.062(0.228 to 4.951)	0.939	16/32(50.0%)		2.488(0.423 to 14.634)	0.313
II	4/5(80.0%)		2.392(0.190 to 30.050)	0.499	3/3(100%)		–	0.999	1/2(50.0%)		1.289(0.040 to 42.040)	0.886
*Clinical T stage*												
T3	19/30(63.3%)	0.051	Ref		11/13(84.6%)	0.113	Ref		8/17(47.1%)	1.000	Ref	
T4	30/55(54.5%)		0.694(0.237 to 2.037)	0.507	17/28(60.7%)		0.321(0.048 to 2.137)	0.240	13/27(48.1%)		0.896(0.191 to 4.214)	0.890
NA	14/16(87.5%)	–	3.275(0.431 to 24.902)	0.252	14/16(87.5%)	–	1.774(0.129 to 24.313)	0668	0			
*Neoadjuvant therapy*												
Anti-PD1 monotherapy	20/28(71.4%)	0.222	Ref		15/19(78.9%)	0.923	Ref		5/9(55.6%)	0.495	Ref	
Anti-PD1 and Anti-CTLA4	11/15(73.3%)		0.783(0.148 to 4.137)	0.773	8/11(72.7%)		0.504(0.053 to 4.811)	0.552	3/4(75.0%)		7.845(0.194 to 317.382)	0.275
Anti-PD1 with chemo- or targeted therapy	32/58(55.2%)		0.597(0.210 to 1.694)	0.332	19/27(70.4%)		0.632(0.141 to 2.827)	0.548	13/31(41.9%)		0.616(0.119 to 3.199)	0.564
*Duration of Neoadjuvant therapy*												
≤ 3 months	35/54(64.8%)	0.781	Ref		19/26(73.1%)	0.927	Ref		16/28(57.1%)	0.238	Ref	
3–6 months	18/29(62.1%)		0.833(0.284 to 2.445)	0.740	14/18(77.8%)		1.212(0.244 to 6.013)	0.814	4/11(36.4%)		0.669(0.137 to 3.276)	0.620
> 6 months	10/18(55.6%)		0.518(0.150 to 1.788)	0.298	9/13(69.2%)		0.889(0.174 to 4.536)	0.888	1/5(20.0%)		0.176(0.013to 2.380)	0.191

*CRC* colorectal cancer, *GC* gastric cancer, *Ref* reference, *pCR* pathological complete response

^a^There were two patients diagnosed with both rectal cancer and right-side colon cancer at the same time, and the treatment factors were recorded separately

**Fig. 2:**
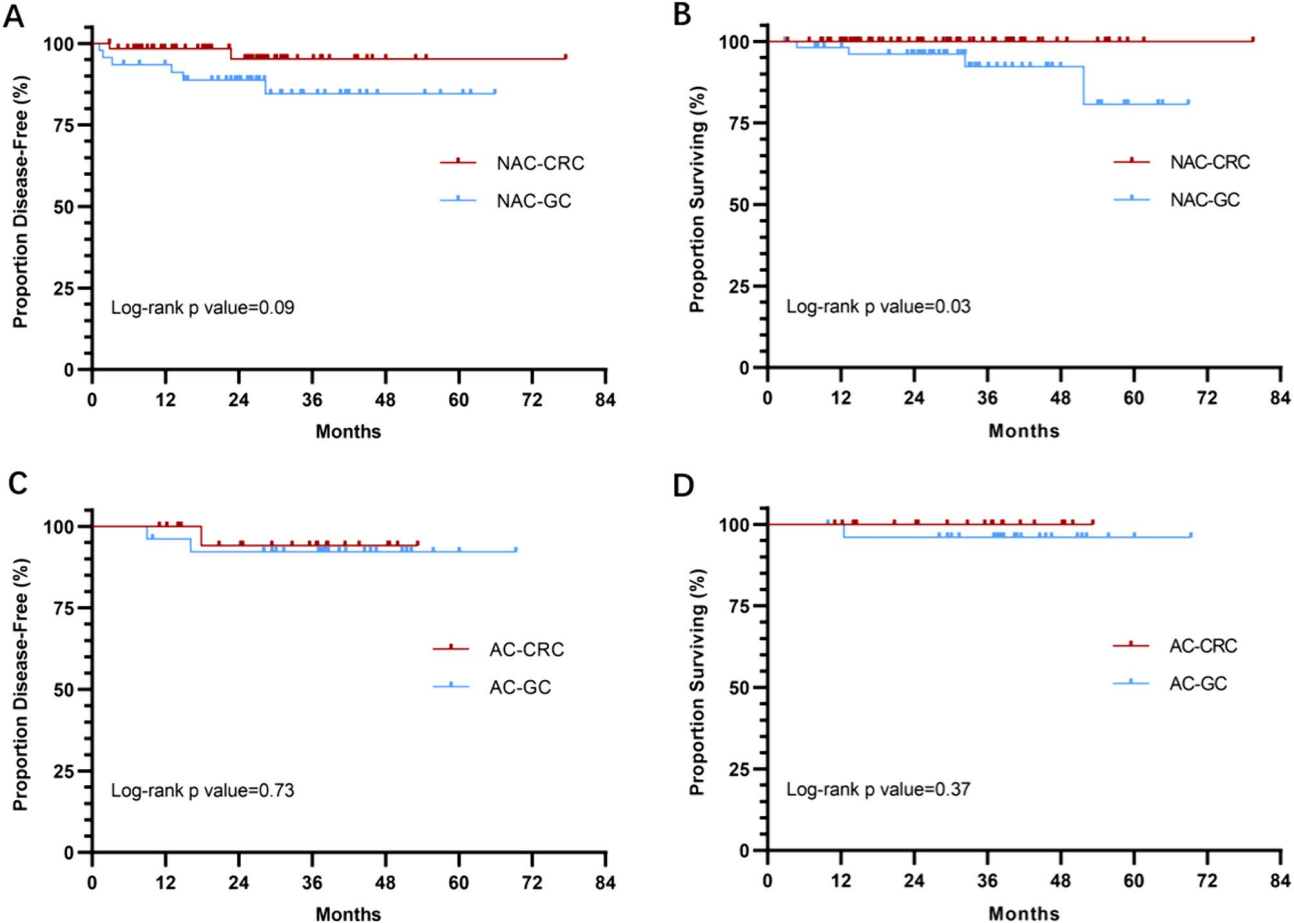
Kaplan Meier curves of disease-free survival (DFS) and overall survival (OS) in neoadjuvant cohort (NAC) and adjuvant cohort (AC). A, DFS of colorectal cancer (CRC) (*n* = 62) and gastric cancer (GC) (*n* = 46) in NAC, analyzed among patients undergoing surgery or achieving clinical complete response. B, OS of CRC (*n* = 68) and GC (*n* = 56) in NAC, analyzed among all patients. C and D, DFS (C) and OS (D) of CRC (*n* = 22) and GC (*n* = 26) in AC, analyzed among all patients

The 2-year DFS (2y-DFS) and 3-year DFS (3y-DFS) rates were 96% (95% CI 90–100%) and 96% (95% CI 90–100%) for CRC, and 88% (78–98%) and 84% (72–96%) for GC (Fig. 2A). The 2-years OS (2y-OS) and 3-year OS (3y-OS) rates were 100% and 100% for CRC, and 96% (90–100%) and 93% (85–100%) for GC (Fig. 2B). Although there were no statistically significant differences, patients who achieved pCR tended to have longer DFS and OS than those did not achieve, especially for GC (Supplementary Figs. 1 and 2). For patients with clinical stage IV, the OS and DFS are numerically inferior to those with non-stage IV (Supplementary Figs. 1 and 3).

### Adjuvant group

#### Patient characteristics

Forty-eight patients were enrolled finally. At data cut-off, the median follow-up time were 35.5 months (11.0–53.2 months) for CRC and 40.4 months (9.9–69.3 months) for GC. The median age for CRC was 42.5 years (ranging from 18 to 71 years), younger than that for GC (median 59.5 years, ranging from 39 to 69 years). CRC patients were more likely to suffer from Lynch syndrome than GC patients (Table 1). Most of patients (> 80%) received anti-PD1 antibody monotherapy as adjuvant treatment (Supplementary Tables 1 and 2).

#### Efficacy

The 2y-DFS and 3y-DFS rates were 94% (84–100%) and 94% (84–100%) for CRC, and 92% (82–100%) and 92% (82–100%) for GC (Fig. 2C). The 2y-OS and 3y-OS rates were 100% and 100% for CRC, and 96% (88–100%) and 96% (88–100%) for GC (Fig. 2D).

### Patients with relapse

In NAC, two CRC patients and five GC patients had tumor relapse after radical resection, and among patients who did not receive surgery, only one GC patient (case 11) had tumor progression 45 months later after stopping ICIs and died of COVID-19 (Supplementary Table 3). Case 3 had a pMMR GC tumor which was confirmed as MSI-H with tumor mutation burden (TMB) of 58 Muts/Mb using biopsy sample from the primary tumor. The pathological stage was ypT3N0 with TRG of 3 after ICI-based neoadjuvant treatment. Immunohistochemistry and NGS were performed again using the surgery sample and it was found that the tumor turned to be pMMR and MSS with TMB of 8 Muts/Mb. Similarly, the primary tumor of case 7 (GC) was dMMR/MSI-H at initial diagnosis, but turned to pMMR after treatment. Case 4 (mixed adeno-neuroendocrine carcinoma of stomach) had only negative MSH2 expression and no PCR or NGS was performed to validate the MSI status. Case 5 (GC) progressed on neoadjuvant nivolumab plus chemotherapy and received salvage radical resection, and liver metastasis occurred soon after surgery. In AC, three patients (1 CRC and 2 GC) had tumor recurrence or metastasis, and one GC patients died of cancer. Among the seven patients with distant metastases, four received dual blockade of PD1 and CTLA4 combined with or without chemo- and targeted drugs, with three PR and one PD (Supplementary Table 3).

## Discussion

To our best knowledge, this is the first study reporting the promising long-term survivals of neoadjuvant ICIs and adjuvant ICIs in dMMR/MSI-H CRC and GC with the longest follow-up period.

More and more efficacy data supported the use of neoadjuvant ICIs in patients with dMMR/MSI-H CRC and GC. However, long-term survival benefits remained unclear. With a median follow-up of 17.2 months in CRC patients treated with neoadjuvant PD1 antibody plus other treatment, the 2y-DFS rate was 100% in a retrospective study [18]. For GC patients treated with dual blockade of PD1 and CTLA4, no relapse was observed with a median follow-up of 13.4–14.9 months in NEONIPIGA study [15]. INFINITY study showed that disease recurred in 2 of 18 GC patients treated with dual blockade of PD1 and CTLA4 with a follow-up of 13.4 months (9.7–14.2 moths)[14]. In this study, the follow-up time was 29.4 and 33.0 months for CRC and GC respectively, much longer compared with other reports, and our data showed 3y-DFS were 96% and 84%, respectively. Notably, a total of 30 (23.8%) patients with stage IV disease were included and received neoadjuvant ICIs in our study. Although the long-term survivals of these patients were numerally lower than that of those with non-IV disease, the DFS and OS were exciting. These results indicated that neoadjuvant ICIs could bring promising long survival benefits in non-metastatic as well as resectable metastatic CRC and GC with dMMR/MSI-H.

In this study, patients with non-pCR seemed to have poorer (but not significantly) DFS and OS in NAC, especially in GC. And in a more recent publication, no local regrowth or distant metastasis was observed in 24 patients with clinical complete response with a median follow-up time of 29.1 months [19]. Therefore, it is reasonable to assume that pCR was associated with better survivals, and further study should be conducted to increase the pCR rate. However, there was no convincing data regarding the most appropriate strategy associated with the highest pCR rate,

For CRC, patients with non-metastatic dMMR colon cancer receiving around 6 weeks of dual blockade of PD1 and CTLA4 and 8 weeks of dual blockade of PD1 and LAG3, respectively in the NICHE2 and NICHE3 studies, and the pCR rates were 67% (72/107) and 79%, respectively. The role of PD1 antibody monotherapy had been explored. In the PICC study, 11 out of 17 (65%) patients treated with 3 months of toripalimab achieved pCR. Creck et al. reported that all the 12 patients achieved cCR after 6 months of dostarlimab. Considering all the results above, it seemed that 6 months of PD1 antibody monotherapy might be preferred to achieve the greatest possibility of CR in CRC, which was supported by another prospective study, which demonstrated that the median time to reach a clinical complete response was 5·2 months [13, 20]. In GC, 3 months of PD1 antibody plus CTLA4 antibody was the only regimen investigated with pCR rate of 58.6–60% [14, 15]. PD1 antibody monotherapy was also used [21]. Interestingly, PD1 antibody plus chemotherapy was the most used neoadjuvant regimen in most retrospective studies related to this field [22–24], which was similar in our study, where 47.4% of CRC patients and 70.5% of GC patients received PD1 blockade combined with chemotherapy. The combination with chemotherapy might be not necessary because neoadjuvant chemotherapy was not associated with better prognosis compared with surgery alone in GC [25] and were likely to result in poor response in CRC [26], which was further supported by our data that treatment regimen was not associated with pCR rate. In clinical practice, the decision to add chemotherapy was likely driven by concerns about disease progression with PD1 antibody monotherapy and the high cost and toxicity associated with dual PD1 and CTLA4 blockade. Prospective clinical trials are warrant to explore the most suitable neoadjuvant strategy, in resectable dMMR/MSI-H CRC and GC, in particular to investigate the role of chemotherapy added to ICIs and the treatment duration.

The optimal adjuvant treatment is also controversial for patients with dMMR/MSI-H CRC or GC who did not receive neoadjuvant ICIs. For CRC, chemotherapy of fluoropyridine plus oxaliplatin had been proven to increase OS or DFS in Stage III or pT4N0 CRC [17, 27]. However, the survival outcomes were still not satisfying. Our results firstly reported 3y-DFS of 94% in CRC after adjuvant anti-PD1 antibody (81.8% treated without chemotherapy) with a relatively long follow-up (median 35.5 months), much higher than historical data (3-DFS 75–80% for stage III CRC [17]). These data highlighted the potential benefits of ICIs in adjuvant setting. Clinical trials are needed to confirm the superiority of anti-PD1 antibody with or without chemotherapy over chemotherapy alone.

Regrading GC, dMMR/MSI-H status might predict lack of benefit in DFS and OS of adjuvant chemotherapy in a multinational, individual-patient-data meta-analysis [16]. Moreover, most retrospective studies did not demonstrate a significant improvement in survivals with the implementation of adjuvant chemotherapy [28]. However, no alternative treatment was proposed for this population. Our study firstly reported the 3y-DFS of 92% after adjuvant ICIs in a relatively late-stage disease (65.4% stage III and 11.5% stage IV), which was numerally higher than that with surgery only or postoperative chemotherapy (~ 80%) [16]. This study might provide a potential treatment option for dMMR/MSI-H GC, in spite of the small sample and mixed therapies used. The ongoing clinical trials will give us more data about the necessity of chemotherapy and the value of ICIs in the adjuvant setting (NCT05236972, NCT05468138, NCT04969029).

Few relapses had been reported in patients with dMMR/MSI receiving perioperative ICIs [13, 14, 18], and the reasons for relapse remain largely unknown. Although our study observed limited cases of relapse, they may offer insights into the underlying mechanisms. Two GC patients (cases 3 and 7) exhibited different MMR/MSI statuses between pre-treatment and post-treatment samples. This could be attributed to MMR/MSI heterogeneity, where tumor cells with dMMR/MSI-H were eliminated after ICIs and only pMMR/MSS cells remained. We suspect that the recurrent tumors were also pMMR/MSS; however, these two patients declined re-biopsy. One patient (case 4) had negative MSH2 expression and positive MSH6 expression, which is uncommon as methylation or mutations in *MSH2* are typically associated with IHC loss of both MSH2 and MSH6 [29], raising suspicion that the tumor was not MSI-H; however, no PCR or NGS tests were performed. Additionally, one patient (case 5) underwent salvage surgery after progression on ICIs developed liver metastasis soon after surgery. Therefore, MMR/MSI heterogeneity, potential wrong assessment of MMR/MSI and resistance to neoadjuvant ICIs might be associated with tumor relapse. Seven patients with relapse had distant metastases and 4 of them received dual blockade PD1 and CTLA4 with or without chemo- and targeted therapy, with 3 PR and 1 PD. These findings supported that rechallenge with ICIs plus other drugs was effective, consistent with our previous retrospective study [30]. We are conducting a clinical trial to investigate the efficacy of intensive ICIs plus anti-VEGF treatment beyond progression on monotherapy PD1 antibody (NCT06099821).

This study has some limitations. As a retrospective study, the heterogeneity among patients, stages, treatment regimen and duration is the major limitation of our study; however, the application of ICIs in neoadjuvant and/or adjuvant period did improve the prognosis of dMMR/MSI-H patients. Another limitation is the small number of patients enrolled due to the relatively lower prevalence of dMMR/MSI-H, especially for the adjuvant use of ICIs. Though promising and novel, our data need to be validated in a prospective study with larger sample.

In conclusion, this study confirmed the survival benefits of neoadjuvant ICIs with the longest follow-up ever reported and provided the first evidence of adjuvant ICIs in dMMR/MSI-H CRC and GC.

### Supplementary Information

Below is the link to the electronic supplementary material.

Supplementary file1 (PDF 603 KB)

Supplementary file2 (PDF 359 KB)

## Data Availability

The authors confirm that the data supporting the findings of this study are available within the article and its supplementary materials.

## Abbreviations

AC: Adjuvant cohort
CRC: Colorectal cancer
CTLA4: Cytotoxic T lymphocyte-associated antigen-4
DFS: Disease-free survival
dMMR: Deficiency of mismatch repair
GC: Gastric cancer
GI: Gastrointestinal
IC: Intestinal cancer
ICIs: Immune checkpoint inhibitors
MSI-H: Microsatellite instability-high
MSS: Microsatellite stability
NAC: Neoadjuvant cohort
NGS: Next-generation sequencing
OS: Overall survival
pCR: Pathologically complete response
PCR: Polymerase chain reaction
PD: Progressive disease
PD1: Programmed cell death 1 protein
pMMR: Proficient mismatch repair
PR: Partial response
TRG: Tumor regression grade
TMB: Tumor mutation burden
