# Supplementary material for: Long-term survivals of immune checkpoint inhibitors as neoadjuvant and adjuvant therapy in dMMR/MSI-H colorectal and gastric cancers
Source: Cancer Immunol Immunother. 2024 Jul 5;73(9):182. doi: 10.1007/s00262-024-03764-9 (PMC11226604; doi:10.1007/s00262-024-03764-9)
Supplement: Supplementary file 1 — Supplementary file1 (PDF 603 KB) [file 262_2024_3764_MOESM1_ESM.pdf]

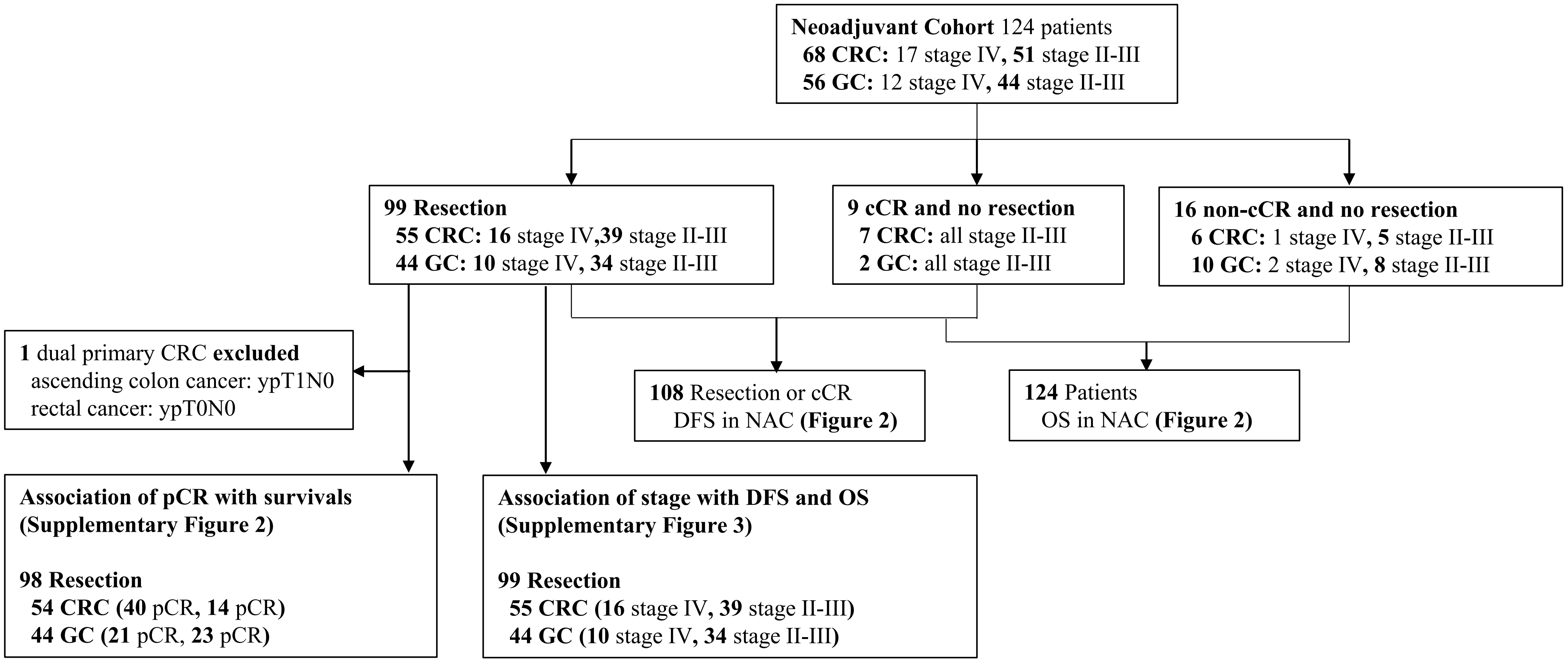

Supplementary Figure 1: Flow of survival analysis in Neoadjuvant Cohort.
Abbreviations: CRC: colorectal cancer; GC: gastric cancer; cCR: clinical complete response; pCR: pathological complete response; DFS: disease-free survival; OS: overall survival

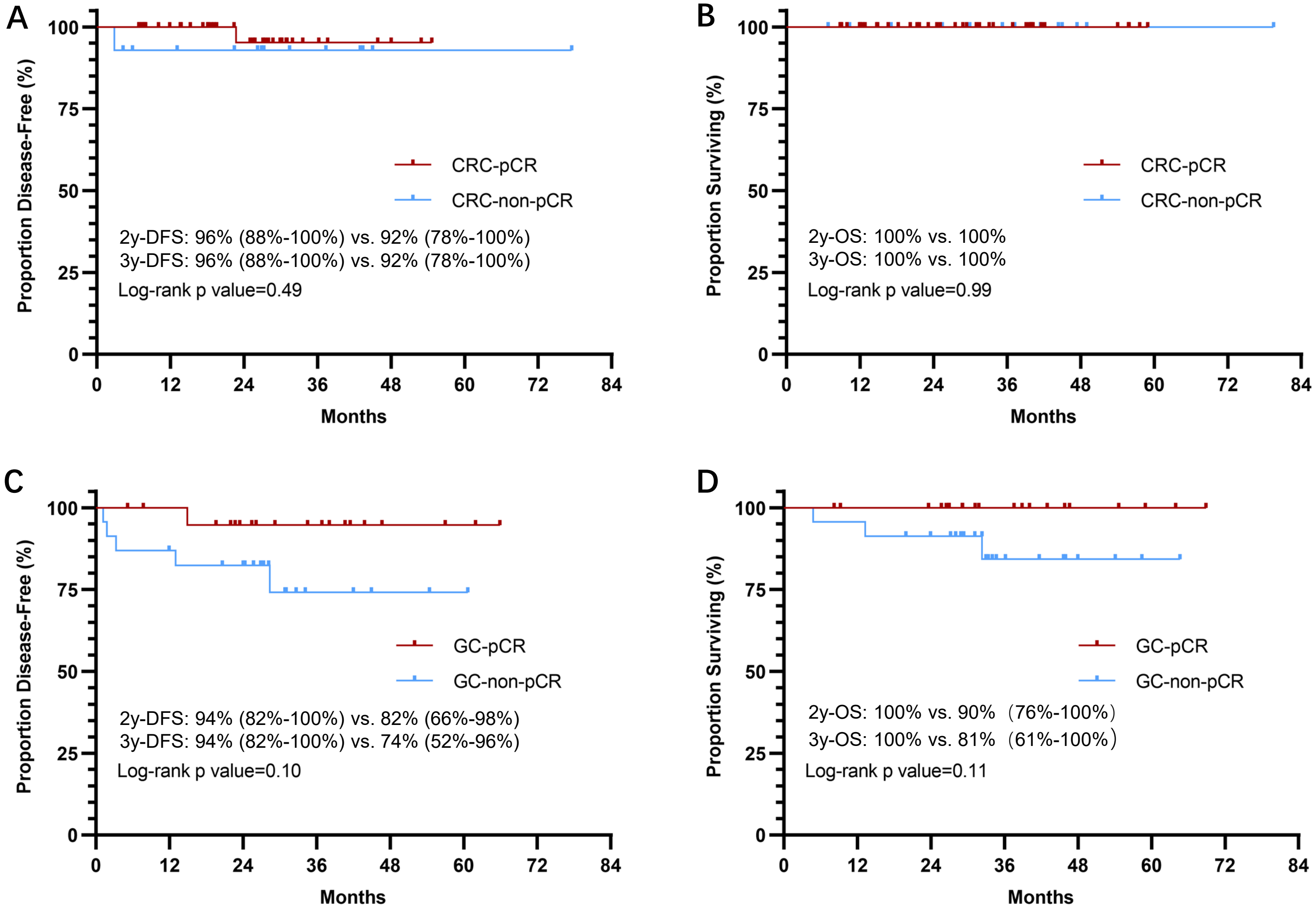

Supplementary Figure 2: Kaplan Meier curves of disease free survival (DFS) and overall survival (OS) according to the pathological response in neoadjuvant cohort (NAC), analyzed among patients undergoing surgery except 1 patients with dual primary CRC who could not be categorized due to different pathological response (ascending colon cancer ypT1N0, rectal cancer ypT0N0).

A and B, DFS (A) and OS (B) of pCR (n=40) and non-pCR (n=14) of CRC. C and D, DFS (C) and OS (D) of pCR (n=21) and non-pCR (n=23) of GC.

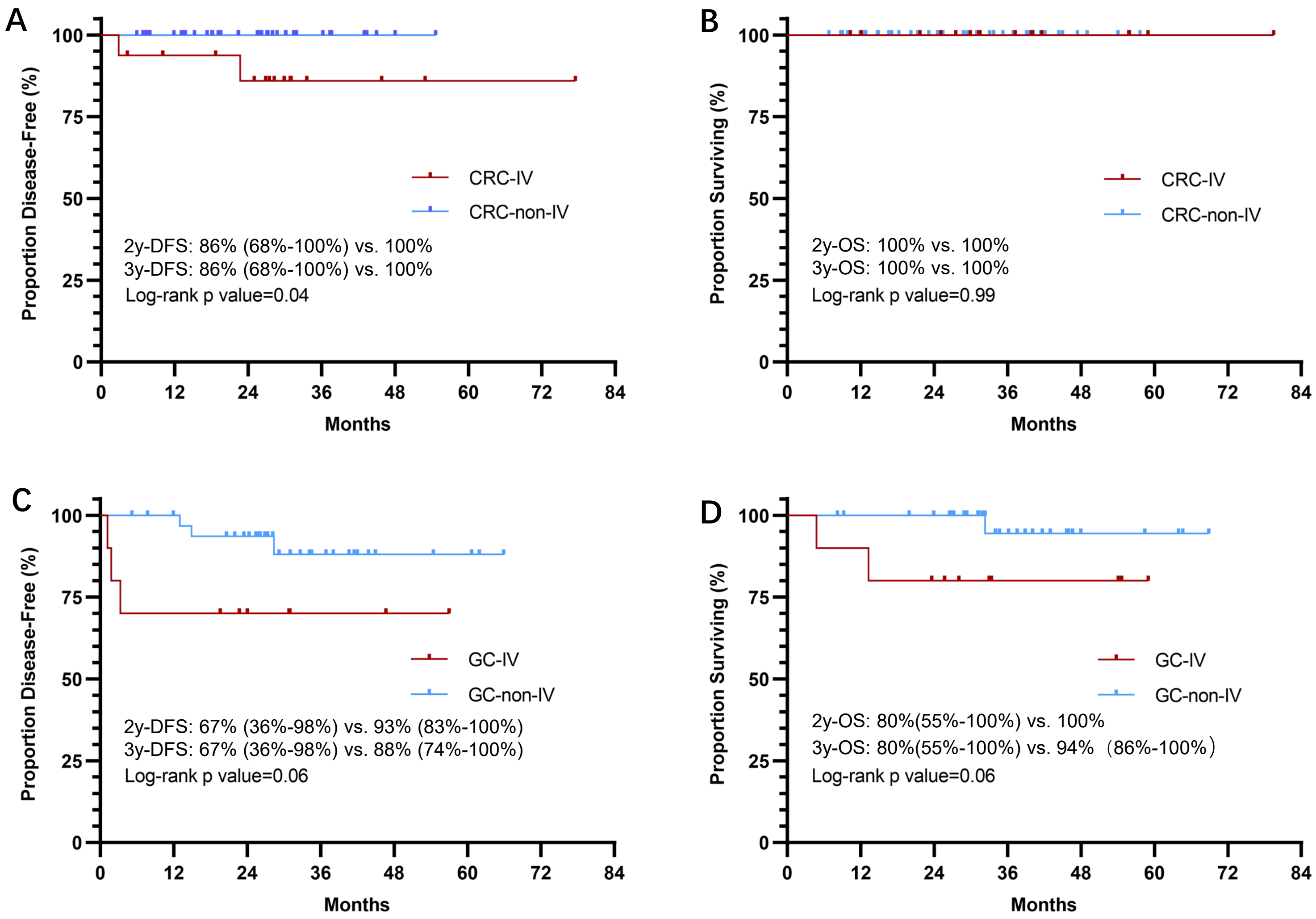

Supplementary Figure 3. Kaplan Meier curves of disease free survival (DFS) and overall survival (OS) according to clinical stages in neoadjuvant cohort (NAC), analyzed among patients undergoing surgery.
A and B, DFS (A) and OS (B) of stage IV (n=16) and stage II-III (n=39) of CRC. C and D, DFS (C) and OS (D) of stage IV (n=10) and stage II-III (n=34) of GC.
