# Supplementary material for: Long-term survivals of immune checkpoint inhibitors as neoadjuvant and adjuvant therapy in dMMR/MSI-H colorectal and gastric cancers
Source: Cancer Immunol Immunother. 2024 Jul 5;73(9):182. doi: 10.1007/s00262-024-03764-9 (PMC11226604; doi:10.1007/s00262-024-03764-9)
Supplement: Supplementary file 2 — Supplementary file2 (PDF 359 KB) [file 262_2024_3764_MOESM2_ESM.pdf]

Supplementary Table 1. Treatment characteristics of Neoadjuvant Cohort and Adjuvant Cohort.

Treatment factor	Neoadjuvant Cohort				Adjuvant Cohort			
	Total (*n* = 124)	Colorectal cancer (*n* = 68)	Gastric cancer (*n* =56)	P value	Total (*n* = 48)	Colorectal cancer (*n* = 22)	Gastric cancer (*n* = 26)	P value
**Neoadjuvant therapy**								
Anti-PD1 monotherapy	40(32.3%)	25(36.8%)	15(26.8%)	0.145	NA	NA	NA	NA
Anti-PD1 and Anti-CTLA4	16(12.9%)	11(16.2%)	5(8.9%)		NA	NA	NA	
Anti-PD1 combined with chemo- or targeted therapy	68(54.8%)	32(47.1%)	36(64.3%)		NA	NA	NA	
None	NA	NA	NA		48(100.0%)	22(100.0%)	26(100.0%)	
**Duration of Neoadjuvant therapy**								
≤3 months	59(47.6%)	28(41.2%)	31(55.4%)	0.288	NA	NA	NA	NA
3-6 months	37 (29.8%)	23(33.8%)	14(25.0%)		NA	NA	NA	
> 6 months	28 (22.6%)	17(25.0%)	11(19.6%)		NA	NA	NA	
**Radical surgery**								
Yes	99(79.8%)	55(80.9%)	44(78.6%)	0.750	48(100.0%)	22(100.0%)	26(100.0%)	NA
No	25(20.2%)	13(19.1%)	12(21.4%)		NA	NA	NA	
**Adjuvant therapy for surgical patients**	**(*n* = 99)**	**(*n* = 55)**	**(*n* =44)**					
Anti-PD1 monotherapy	31(31.3%)	20(36.4%)	11(25.0%)	0.003	40(83.3%)	18(81.8%)	22(84.6%)	1.000
Anti-PD1 combined with chemotherapy or targeted therapy	11(11.1%)	1(1.8%)	10(22.7%)		8(16.7%)	4(18.2%)	4(15.4%)	

Chemotherapy ±targeted therapy	4(4.0%)	1(1.8%)	3(6.8%)		NA	NA	NA	
Observation	53(53.5%)	33(60.0%)	20(45.5%)		NA	NA	NA	
**Duration of Adjuvant therapy for surgical patients**	**(*n* = 99)**	**(*n* = 55)**	**(*n* =44)**					
≤6 months	32(32.3%)	15(27.3%)	17(38.6%)	0.213	22(45.8%)	11(50.0%)	11(42.3%)	0.369
6-12 months	12(12.1%)	5(9.1%)	7(15.9%)		20(41.7%)	7(31.8%)	13(50.0%)	
> 12 months	2(2.0%)	2(3.6%)	0		6(12.5%)	4(18.2%)	2(7.7%)	
None or unknown	53(53.5%)	33(60.0%)	20(45.5%)		NA	NA	NA	

Abbreviations: NA: not appliable.

Supplementary Table 2. Treatment regimen details of neoadjuvant therapy in Neoadjuvant Cohort and adjuvant therapy in Adjuvant Cohort

Treatment regimens	Neoadjuvant Cohort			Adjuvant Cohort		
	Total (*n* = 124)	Colorectal cancer (*n* = 68)	Gastric cancer (*n* = 56)	Total (*n* =48)	Colorectal cancer (*n* = 22)	Gastric cancer (*n* = 26)
**Anti-PD1 monotherapy**	40(32.6%)	25(36.8%)	15(26.8%)	40(83.3%)	18(81.8%)	22(84.6%)
**Anti-PD1+ ipilimumab**	16(12.9%)	11(16.2%)	5(8.9%)	0	0	0
**Anti-PD1+ chemotherapy and/or targeted therapy**	68(54.8%)	32(47.1%)	36(64.3%)	8(16.7%)	4(18.2%)	4(15.4%)
XELOX	17(13.7%)	8(11.8%)	9(16.1%)	4(8.3%)	4(18.2%)	0
SOX	17(13.7%)	0	17(30.4%)	4(8.3%)	0	4(15.4%)
FOLFOX	1(0.8%)	0	1(1.8%)	0	0	0
FOLFIRI	2(1.6%)	1(1.5%)	1(1.8%)	0	0	0
FOLFOXIRI	2(1.6%)	1(1.5%)	0	0	0	0
FLOT	4(3.2%)	0	4(7.1%)	0	0	0
S-1	1	0	1			
Nab-PTX	2(1.6%)	0	2(3.6%)	0	0	0
IRI	1(0.8%)	1(1.5%)	0	0	0	0
IRI+Bev/Cet	13(10.5%)	12(17.6%)	0	0	0	0
XELOX+Bev	2(1.6%)	2(2.9%)	0			
Bev/Fruquintinib/Lenvatinib	8(6.5%)	7(10.3%)	1(1.8%)	0	0	0

Abbreviations: XELOX: capecitabine + oxaliplatin; SOX: Tegafur, Gimeracil and Oteracil Potassium + oxaliplatin; FOLFOX: 5-fluorouracil + oxaliplatin; FOLFIRI: 5-fluorouracil + irinotecan; FOLFOXIRI: 5-fluorouracil + oxaliplatin + irinotecan; FLOT: 5-fluorouracil + oxaliplatin + docetaxel; S-1: Tegafur, Gimeracil and Oteracil Potassium; Nab-PTX: Albumin paclitaxel; IRI: irinotecan; Bev: Bevacizumab; Cet: Cetuximab.

Supplementary Table 3. The details of relapsed cases in Neoadjuvant Cohort and Adjuvant Cohort

No.	Age (y)	Sex	Tumor type	Cohort	Initial stage	MMR/MSI/TMB	Neoadjuvant	Surgery sample		Adjuvant	DFS (m)	Relapse site	First-line treatment after relapse			OS (m)
								(y)pTNM	MMR/MSI/TMB				Regimen	Response	PFS(m)	
1	35	F	CRC	NAC	IV	MSH2/MSH6 (-), PCR-MSI-H	Anti-PD1 +TT, 4.0m, PR	ypT3N0M0	MSH2/MSH6 (-) PCR/NGS not performed	Anti-PD1, 2.0m	2.9	Distant lymph node	Anti-PD1 + Anti-CTLA4	PR	*27.8+	37.2*
2	42	F	CRC	NAC	IV	MLH1/PMS2 (-), PCR-MSI-H	Anti-PD1 +CT, 2.0m, PR	pCR	NA	None	22.7	Peritoneum	Not started	NA	NA	25.1*
3	71	M	GC	NAC	IV	pMMR, NGS-MSI-H, NGS-TMB 58Muts/Mb	Anti-PD1 +TT, 6.0m, SD	ypT3N0M0	pMMR NGS-MSS NGS-TMB 8Mubs/Mb	CT+TT, 1.0m	1.8	Lung	CT + TT	SD	23.5	54.1*
4	50	M	GC	NAC	III	MSH2 (-), PCR/NGS not performed	Anti-PD1 +CT, 4.0m, SD	ypT4N0M0	MSH2 (-) PCR/NGS not performed	Anti-PD1 +CT, 4.0m	28.3	Death of unknown reason	NA	NA	NA	32.3
5	70	M	GC	NAC	IVA	MLH1/PMS2 (-), NGS-MSI-H, NGS-TMB 64Muts/Mb	Anti-PD1 +CT, 2.0m, PD	ypT3N2M0	MLH1/PMS2 (-) PCR/NGS not performed	None	1.2	Liver	Unknown	NA	NA	13.3
6	69	F	GC	NAC	III	MLH1/PMS2 (-), PCR/NGS not performed	Anti-PD1 +CT, 5.0m, PR	pCR	NA	Anti-PD1, 12.0m	14.9	Peritoneum	AK104 + AK109+PTX	PR	*10.7+	31.8*
7	71	M	GC	NAC	III	MLH1/PMS2 (-), PCR-MSI-H	Anti-PD1 +CT, 4.0m, SD	ypT4N3M0	MLH1 (-), PCR/NGS not performed	None	13.0	Distant lymph node	None	NA	NA	22.9*

8	58	M	CRC	AC	IV	NA	None	pT3N1M1a, synchronous liver metastasis	MLH1/PMS2 (-), NGS-MSI-H, NGS-TMB 65Muts/Mb	Anti-PD1 +CT, 6.0m	17.8	Second colon cancer, cT3N0M0	Anti-PD1 followed by radical surgery, ypT3N0M0	CR	*29.3+	48.2*
9	64	M	GC	AC	III	NA	None	pT4N2M0	MLH1/PMS2 (-), NGS-MSI-H, NGS-TMB 48Muts/Mb	Anti-PD1, 15.0m	16.1	Liver	AK104 + AK109+PTX	PR	*21.7+	40.8*
10	69	F	GC	AC	III	NA	None	pT3N3M0	MLH1/PMS2 (-), PCR-MSI-H	Anti-PD1 +CT, 6.0m	8.9	Liver and distant lymph node	Anti-PD1 + Anti-CTLA4	PD	1.5	12.5
11	78	M	GC	NAC	III	MLH1/PMS2 (-), PCR-MSI-H	Anti-PD1, 5.0m, PR	Refusal of surgery	NA	NA	PFS 50.7m	Peri-gastric lymph node	Anti-PDL1	SD	8.3	63.9

Abbreviations: CT: chemotherapy, TT: targeted therapy, NA: not appliable, PTX: paclitaxel, pCR: pathological complete response, CR: complete response, SD: stable disease, PD: progressive disease, PR: partial response, DFS: disease-free survival, PFS: progressive-free survival, OS: overall survival, AK104: an immune checkpoint inhibitor both anti-PD1 and anti CTLA4, AK109: a monoclonal antibody inhibits vascular endothelial growth factor receptor-2 (VEGFR2). Index: * patients without disease progression, + still in first-line treatment, * alive.
